# Quantitative MRI-based decision model for early-stage parkinsonism diagnosis: a pilot feasibility study

**DOI:** 10.1016/j.ynirp.2025.100273

**Published:** 2025-06-14

**Authors:** Laura Nunez-Gonzalez, Elise G.P. Dopper, Anke W. van der Eerden, Samy Abo Seada, Agnita J.W. Boon, Marcel M. Verbeek, Bastiaan R. Bloem, Frederick Jan Anton Meijer, Juan Antonio Hernandez-Tamames

**Affiliations:** a Department of Radiology and Nuclear Medicine, Erasmus MC, Rotterdam, the Netherlands; b Department of Neurology, Erasmus MC, the Netherlands; c Alzheimer Centre, Erasmus MC, Rotterdam, the Netherlands; d Surgical Reality, Amsterdam, the Netherlands; e Department of Neurology, Radboud University Medical Center, Nijmegen, the Netherlands; f Department of Human Genetics, Radboud University Medical Center, Nijmegen, the Netherlands; g Department of Medical Imaging, Radboud University Medical Center, Nijmegen, the Netherlands; h Department of Imaging Physics, TU Delft, the Netherlands

## 1 Introduction

Parkinsonism is a clinical syndrome defined as bradykinesia, combined with rest tremor, rigidity, or both (Postuma et al., 2015). Parkinson's disease (PD) is the most common cause of parkinsonism and the fastest-growing neurodegenerative disorder worldwide with currently almost 12 million affected people worldwide (Bloem et al., 2021; Murray, 2024). Atypical parkinsonisms, including progressive supranuclear palsy (PSP), multiple system atrophy (MSA), corticobasal syndrome (CBS), and dementia with Lewy bodies (DLB), are collectively less prevalent than idiopathic PD. These disorders are classified as rare, with estimated prevalence rates ranging from 5 to 22 cases per 100,000 population depending on the specific subtype and diagnostic criteria used (Lo, 2022). MSA and PSP are the most frequently encountered atypical forms. For example, MSA has a reported prevalence of about 3–5 per 100,000, while PSP may reach up to 6 per 100,000 in some studies. DLB, often overlapping with both PD and Alzheimer's disease, appears to be more common, with estimates around 0.4 %–5 % of the elderly population, depending on whether clinical or neuropathological criteria are used (Nysetvold et al., 2024; Sekiya et al., 2024; Delpirou et al., 2024). Diagnosis is typically made based on clinical grounds, and several exclusion criteria as well as red flags have been defined that should urge the clinician to consider an atypical parkinsonian syndrome (Postuma et al., 2015). For instance, in case of early severe autonomic failure or frequent falls, the diagnosis of MSA or PSP should be considered respectively (Wenning et al., 2022; Höglinger et al., 2017). Atypical parkinsonism (AP) has a more aggressive disease course than PD, leading to earlier loss of independent functioning and shorter life spans. Moreover, dopamirgenic treatments are less effective when applied in persons with AP. Therefore, for appropriate guidance and treatment, a timely accurate diagnosis is crucial. However, AP diagnoses are frequently missed in the early stages with reported sensitivities for MSA and PSP below 65 % (Hughes et al., 2002; Joutsa et al., 2014).

The latest diagnostic criteria for MSA and PSP have incorporated MRI findings. Prominent midbrain atrophy is considered a supportive feature of PSP (Höglinger et al., 2017). Atrophy of the putamen, middle cerebellar peduncle, pons or cerebellum, a “hot-cross bun” sign, putaminal signal decrease on iron-sensitive images, and increased diffusivity of putamen or middle cerebellar peduncle are defined as MRI markers for clinically established MSA (Wenning et al., 2022). However, these MRI characteristics often manifest only late in the disease course, thereby hampering their usefulness for early diagnostics. For MSA, adding the MRI markers to diagnostic criteria even seems to have reduced sensitivity without increasing specificity (Jensen et al., 2024).

Quantitative MRI techniques showed promising results in enhancing the diagnostic accuracy (Heim et al., 2017; Chougar et al., 2020; Lehericy et al., 2017; Pyatigorskaya et al., 2020a; Bae et al., 2021), however, they have not been extensively tested in large-scale studies at an early-stage. The clinical adoption of advanced techniques like quantitative susceptibility mapping (QSM) and diffusion tensor imaging (DTI) is hindered by challenges in standardization (Jensen et al., 2024), inconsistent performance, and implementation difficulties (Wang et al., 2017).

Although several quantitative MRI biomarkers have shown potential in differentiating PD from disorders such as MSA or PSP, no single MRI biomarker has reliably distinguished between them. A potential solution could involve combining multiple quantitative MRI biomarkers into a decision model (Bae et al., 2021), as proposed in recent research (Pyatigorskaya et al., 2020b; He et al., 2021; Jin et al., 2019; Leng et al., 2022). Abo Seada et al. (2023) proposed a decision tree made up of decision rules which uses atrophy measurements in and around the brainstem (Oba et al., 2005; Nicoletti et al., 2006; Quattrone et al., 2008; Massey et al., 2013) and of the third ventricle (Quattrone et al., 2018), QSM markers (Mazzucchi et al., 2019; Sjöström et al., 2017), DTI markers (Du et al., 2017), and neuromelanin MRI of the substantia nigra (SN) and locus coeruleus (LC) (Ohtsuka et al., 2014).

In this work, we applied the proposed pipeline (Seada et al., 2023) retrospectively to 73 patients with a final clinical diagnosis of PD, MSA or PSP to evaluate the performance and feasibility of MRI-biomarkers in differentiating between PD, MSA, and PSP at early disease stages. These patients were included when the clinical diagnosis was still uncertain.

## 2 Methods

### 2.1 Population

For this study, we used data that were originally prospectively acquired for a different research project (Meijer et al., 2015; Van et al., 2018). Between 2010 and 2012 patients with parkinsonism but uncertainty about the clinical diagnosis (CUP) and with a disease duration <3 years were included at Radboud University Medical Center - Radboudumc - (Nijmegen, the Netherlands) and underwent clinical evaluation and MRI. Patients were excluded if they already had a clinically probable syndrome, were younger than 18 years, had undergone prior brain surgery, or had another neurodegenerative disorder or unstable comorbidity. Further details on the inclusion and exclusion criteria can be found in (Van et al., 2018). The probable diagnoses (used in this study to classify the patients) were obtained after clinical follow-up (3–12 years later).

All patients provided written informed consent and the Institutional Medical Ethics Committee approved the use of the data for this study.

### 2.2 Clinical evaluation

At the time of inclusion, all patients underwent a thorough neurological examination by a movement disorder specialist (Van et al., 2018). Motor symptoms were rated according to the Movement Disorder Society Unified Parkinson's disease rating scale (MDS-UPDRS) and clinical disease severity was assessed with the Hoehn and Yahr scale (HY) (Goetz et al., 2008). Cognitive screening was performed with the Mini-mental state examination (MMSE) (Folstein et al., 1975).

### 2.3 Clinical follow-up

All patients received clinical follow-up as standard clinical practice. For the current analyses, we used the data from all patients with an initial CUP diagnosis. However, by the time of the current analyses they had a final clinical diagnosis of PD, PSP, or MSA (either the Parkinsonian type (MSA-P), the cerebellar type (MSA-C) or unclassified MSA (MSA-u). Clinical diagnoses were made according to current clinical diagnostic criteria (Postuma et al., 2015; Wenning et al., 2022; Höglinger et al., 2017) by neurologists specialized in movement disorders. The average time between inclusion and final diagnosis (and its standard deviation) was 27 (13) months for PD, 23 (13) months for MSA and 35 (15) months for PSP. All the details per patient are included in Supplementary Tables S1, S2 and S3.

### 2.4 MRI acquisition

MRI scans were acquired on a 3T Siemens MRI scanner (Magnetom Trio; Siemens, Erlangen, Germany) in Radboudumc. The MRI protocol included a 3D high-resolution T1w-SPGR, a 3D multi-echo gradient-echo sequence for susceptibility-weighted image (SWI), and a Diffusion Tensor Image (DTI) sequence. The MRI scan parameters are described in Table 1. For further explanation of these parameters, see Meijer et al. (2015). All the MRI scans were performed shortly after the inclusion of the patients in the study.

**Table 1:** MRI scanning parameters—iPAT indicates integrated parallel acquisition technique.

Sequence	**TR (ms)**	**TE (ms)**	**Flip** **Angle**	**Voxel size (mm)**	IPAT Factor	Acquisition Time (min:sec)	Others
T1w	2300	4.7	12°	1.0x1.0x1.0	2	5:47	TI = 1100 ms
SWI	29	20.0	15°	0.6x0.6x3.0	2	4:42	–
DTI	13000	102.0	90°	2.0x2.0x2.0	2	7:24	# Directions = 30 Diffusion values: b = 0 and b = 1000

### 2.5 MRI-based decision model

We used the acquired MRI sequences following the guidelines in (Seada et al., 2023) to obtain the biomarkers specified in Table 2.

**Table 2:** Specific biomarkers obtained from the MR images:^a^Obtained from T1 weighted scan; ^b^Obtained from diffusion tensor imaging (DTI); ^c^Obtained from susceptibility-weighted image (SWI).

Biomarker	Description
MRPI^*a*^	Magnetic Resonance Parkinsonian Index: multiply pons-to-midbrain ratio with the ratio of the mean diameters of the middle cerebral peduncle (MCP) to the mean diameters of the superior cerebellar peduncles (SCP) (Quattrone et al., 2008)
MRPI2^*a*^	Modification of the MRPI by multiplying it by the ratio of the third ventricle to the frontal horns (Quattrone et al., 2018)
T1 PM_ratio^*a*^	Pons-to-midbrain ratio (Möller et al., 2017)
DTI FA SCP^*b*^	Average fractional anisotropy in the superior cerebellar peduncles (Du et al., 2017; Planetta et al., 2016; Nicoletti et al., 2008)
DTI FA CB^*b*^	Average fractional anisotropy in the cerebellum (Du et al., 2017)
DTI FA MCP^*b*^	Average fractional anisotropy in the middle cerebral peduncle (Du et al., 2017; Worker et al., 2014)
DTI FA PUT^*b*^	Average fractional anisotropy in the putamen (Du et al., 2017; Worker et al., 2014)
SWI PUT^*c*^	Average value of the SWI in the putamen normalized to the SWI mean in the white matter (Mazzucchi et al., 2019; Sjöström et al., 2017; Meijer et al., 2015; Gupta et al., 2010)
SWI STN^*c*^	Average value of the SWI in the substantia nigra normalized to the SWI mean in the white matter (Mazzucchi et al., 2019; Sjöström et al., 2017; Meijer et al., 2015; Gupta et al., 2010)
SWI RN^*c*^	Average value of the SWI in the red nucleus normalized to the SWI mean in the white matter

**Fig. 1:**
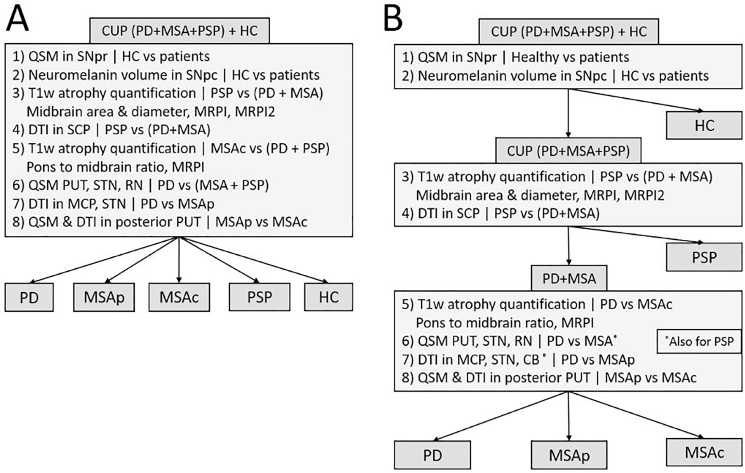
Figure and caption from Seada et al. (Seada et al., 2023): “Decision models for differentially diagnosis Parkinson's disease and other parkinsonisms. A shows a model where all biomarkers are considered in a single multi-class classification. The decision tree in B is made up of two binary classifications followed by one multi-class classification. The cut-off values are weighted combinations of individual decision rules. (∗) indicates metrics that overlap between MSA and PSP patients, and thus rely on separating PSP patients in the second stage. CUP: clinically unclassifiable parkinsonism. PD: Parkinson's disease. MSA: multiple system atrophy. PSP: progressive supranuclear palsy. HC: healthy controls. DTI: diffusion tensor imaging. QSM quantitative susceptibility mapping. PUT: putamen. SCP: superior cerebellar peduncle. STN: subthalamic nucleus. RN: red nucleus. MCP: middle cerebellar peduncle. CB: cerebellum. SNpr: substantia nigra pars reticulata. SNpc: substantia nigra pars compacta.”

Subsequently, we used the decision model proposed by Seada et al. (Seada et al., 2023; Mazzucchi et al., 2019; Sjöström et al., 2017; Meijer et al., 2015; Gupta et al., 2010) (Fig. 1) to classify all patients according to the MRI measures.

Since we used data acquired for a different study, some limitations urged us to apply the following deviations from the decision model.

● We disregarded rules 1 and 2 from the decision model since those are used to differentiate healthy individuals from patients. In practice, it is not as difficult to differentiate healthy individuals from those with CUP compared to the diagnostic challenge later in disease. Therefore, the 2D MT-weighted gradient-echo sequence that can be used to measure neuromelanine volumes was not included in the analyses.
● We had to disregard rule 8, which is used to differentiate MSA-C from MSA-P because of the small number of MSA-C patients included (n = 2)
● Because we were unable to retrieve the information needed for QSM, rule 6 was limited to SWI. We considered these susceptibility-weighted images still useful because all the patients were scanned in the same system and with the same parameters, therefore it should be possible to detect differences in iron concentration due to the specific disease (areas with increased mineralization show less signal intensity in the SWI images (Gupta et al., 2010)).

We used HD-Bet (Isensee et al., 2019), FreeSurfer (http://surfer.nmr.mgh.harvard.edu version 7.3.2) recon-all, and brainstem subsegmentation (Iglesias et al., 2015; Bocchetta et al., 2020) to automatically segment the T1 weighted images and FSL (Jenkinson et al., 2012) and RL-atlas (Tyszka et al., 2022) to segment and register the DTI and SWI images. Deep gray matter segmentation for SWI uses UNet approaches from swi-cnn (Beliveau et al., 2021). No manual correction was needed since the output obtained was visually inspected and it was satisfactory for all the subjects.

These biomarkers can be used all at once (model I) or in different steps (model II) (Seada et al., 2023) to classify the disease into PD, MSA, and PSP (Fig. 1).

### 2.6 Statistical analyses

All the analyses were performed using in-house scripts written in Python. Boxplots of the biomarkers were plotted and a one-way ANOVA was calculated to investigate significant differences between groups.

Subsequently, Bayesian Inference based on the decision models I and II was used to estimate each patient's diagnosis as PD, MSA, or PSP. Subsequently, we could calculate each patient's probability of belonging to the PD, MSA, or PSP group based on the values of their biomarkers. The patient was then classified to the diagnosis with the highest probability. Since the groups' sizes were very different, to obtain a better estimation of the sensitivity, specificity, and accuracy of the methods, we re-ran the Bayesian Inference analysis using bootstrapping (Zhu, 1997) by randomly sampling with replacement of 10 subjects from the MSA group and 10 patients from the PD group, besides the 10 subjects from the PSP group. This experiment was run 25000 times and the average and its 95 % confidence interval (Vangel, 1996) of the sensitivity, specificity, and accuracy (for both models) were calculated.

## 3 Results

### 3.1 Population

A total of 93 patients with an initial diagnosis of CUP were included. Of those, 73 patients had a final clinical diagnosis of PD (n = 38), PSP (n = 10), MSA-P (n = 13), MSA-C (n = 2) or MSA-u (n = 10), and thus were included in the imaging analyses. The remaining 20 patients had different clinical diagnoses during follow-up, such as vascular parkinsonism or Lewy body dementia, or they still had uncertain clinical diagnoses by the time of current analyses. Therefore, they were excluded. Demographic and clinical data are summarized in Table 3.

**Table 3:** Mean (and standard deviation) per diagnosis group of Age, Disease Duration (from first symptoms to MRI scan), MMSE score, HY stadium, UPDRS score, and duration of follow-up (from MRI scan to final clinical diagnosis). Number of Female/Male patients in each group is in the third column.

	Age	Gender Female/Male	Disease Duration (months)	MMSE score	HY stadium	UPDRS score	Follow-up (months)
**PD (n=38)**	61.4 (9.5)	16/22	24.6 (15.0)	28.4 (12.8)	1.7 (0.9)	32.4 (17.6)	27.08 (13.32)
**MSA (n=25)**	64.2 (8.2)	9/16	24.3 (14.5)	28.3 (14.4)	2.4 (1.3)	42.5 (22.7)	23.07 (13.90)
**PSP (n=10)**	64.3 (5.25)	3/7	38.0 (18.9)	28.4 (13.8)	2.7 (1.4)	30.7 (19.5)	35.1 (15.77)

**Table 4:** Shows the mean, the standard deviation, and the results of the one-way ANOVA analysis per biomarker. All biomarkers related to the atrophy or SWI showed differences in at least two of the patient's groups. Conversely, for the DTI biomarkers, we found no significant differences between groups.

	PD (n = 38)	MSA (n = 25)	PSP (n = 10)	F-value	P-value	Significant differences
**MRPI**	143.054 (36.019)	142.024 (48.005)	210.761 (56.858)	10.589	<10^−4^	PSP>(PD & MSA)
**MRPI2**	32.964 (13.7359	33.539 (16.816)	59.316 (25.985)	10.397	<10^−3^	PSP>(PD & MSA)
**T1 PM ratio**	3.414 (0.573)	3.421 (0.738)	4.753 (0.927)	16.396	<10^−6^	PSP>(PD & MSA)
**DTI FA SCP**	670.262 (51.586)	638.731 (107.831)	623.115 (71.839)	2.092	0.13	–
**DTI FA CB**	490.666 (44.345)	476.603 (65.668)	482.678 (37.284)	0.562	0.57	–
**DTI FA MCP**	518.756 (62.025)	484.072 (88.165)	529.740 (25.134)	2.450	0.09	–
**DTI FA STN**	527.369 (94.702)	532.173 (72.043)	497.292 (93.808)	0.600	0.55	–
**DTI FA PUT**	430.992 (49.452)	429.342 (49.454)	446.568 (38.18)	0.499	0.60	–
**SWI PUT**	0.925 (0.083)	0.804 (0.118)	0.796 (0.168)	11.442	<10^−4^	PD > MSA > PSP
**SWI STN**	0.941 (0.103)	0.842 (0.141)	0.698 (0.258)	12.055	<10^−4^	PD > MSA > PSP
**SWI RN**	0.921 (0.049)	0.857 (0.111)	0.703 (0.256)	10.289	<10^−3^	PD > MSA > PSP

## 4 MRI biomarkers analyses

Based on the results of the ANOVA, we decided to exclude the DTI biomarkers from the decision model, since there were no significant group differences. Thus, we disregarded rules 4 and 7, and only used rules 3, 5, and 6 in our model.(see Table 4)

**Fig. 2:**
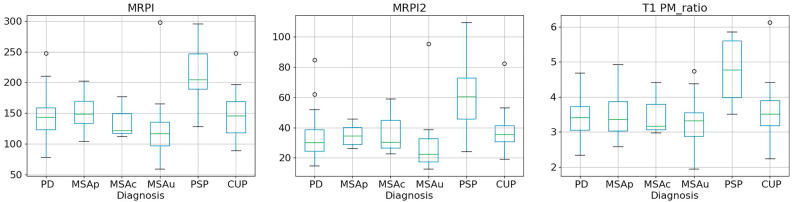
Boxplots of the atrophy biomarkers (MRPI - left -, MRPI2 - middle -, T1 PM_ratio - right -) for Parkinson's disease (PD), multiple system atrophy (MSA) - cerebellar (MSAc), parkinsonian (MSAp) or unclassified (MSAu), progressive supranuclear palsy (PSP), clinically unclassifiable parkinsonism (CUP).

**Fig. 3:**
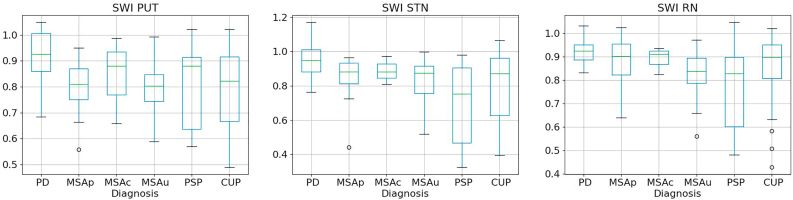
Boxplots of the SWI biomarkers in the putamen (left), substantia nigra (middle) and red nucleus (right) for Parkinson's disease (PD), multiple system atrophy (MSA) - cerebellar (MSAc), parkinsonian (MSAp) or unclassified (MSAu), progressive supranuclear palsy (PSP), and clinically unclassifiable parkinsonism (CUP).

Fig. 2 shows the boxplots for the atrophy biomarkers related to rules 3 and 5. Values for patients with MSA or PD were distinct from those of patients with PSP (rule 3). However, the difference between MSAc patients and PD patients (rule 5) was less obvious. Fig. 3 shows boxplots for the SWI MRI measures used for rule 6 comparing MSA patients versus PD patients. Although the ANOVA showed significant differences between the 3 groups, the boxplots show some overlap between the different patient groups.

## 5 Bayesian Inference

Next, we used Bayesian Inference with the selected biomarking using models I and II to classify each patient as PD, MSA, or PSP. Of the total group, 43 (59 %) and 41 (56 %) patients were correctly classified into their final clinical diagnosis by model I and II respectively.

Table 5 shows the sensitivity, specificity, and accuracy of both models. These parameters are calculated per group, considering in each case the patients included in that group as ‘positives’, and the rest of the patients as ‘negatives’. Both models performed moderately accurately in classifying MSA and PD (accuracy higher than 0.60) and quite well in classifying PSP (accuracy higher than 0.85). Both models have high specificity for diagnosing MSA and PSP with lower sensitivity, while for PD sensitivity and specificity are both around 60 %. Model I seems to perform slightly better than model II, additional details for model I are provided in the Supplementary data.

Subsequently, we used bootstrapping with 25.000 iterations to calculate the average and 95 % confidence intervals for sensitivity, specificity, and accuracy for both models, also shown in Table 5. The estimations reached a steady state around the 15000 iterations (Supplementary Fig. S1).

**Table 5:** Sensitivity, specificity, and accuracy classifying Parkinson's disease (PD), multiple system atrophy (MSA), and progressive supranuclear palsy (PSP) using the classification model I (multi-class with all the biomarkers at once) and the classification model II (two binary classifications, first to separate PSP from the rest and second to separate MSA from PD). Columns 2 to 4 report the results using simple Bayesian inference and columns 5 to 7 report the average (and its 95 % confidence interval) calculated using 25000 iterations with bootstrapping. The sensitivity, specificity and accuracy are calculated per group, considering the specific group as ‘positives’ and the other 2 groups together as ‘negatives’.

	Bayesian Inference			Bayesian Inference with bootstrapping		
*Model 1*	PD (n = 38)	MSA (n = 25)	PSP (n = 10)	PD (n = 38)	MSA (n = 25)	PSP (n = 10)
Sensitivity	0.68	0.44	0.60	0.492 (0.490–0.494)	0.520 (0.518–0.522)	0.615 (0.614–0.616)
Specificity	0.57	0.77	0.93	0.683 (0.682–0.684)	0.748 (0.747–0.749)	0.882 (0.881–0.883)
Accuracy	0.63	0.66	0.89	0.619 (0.618–0.620)	0.672 (0.671–0.673)	0.793 (0.792–0.794)
*Model 2*						
Sensitivity	0.68	0.36	0.60	0.443 (0.441–0.445)	0.443 (0.441–0.445)	0.655 (0.655-, 0.656)
Specificity	0.60	0.77	0.89	0.755 (0.754–0.756)	0.724 (0.723-0.725)	0.826 (0.825,- 0.826)
Accuracy	0.64	0.63	0.85	0.673 (0.672-0.674)	0.630 (0.629-0.631)	0.769 (0.768-,0.769)

### 5.1 Outliers analysis

**Fig. 4:**
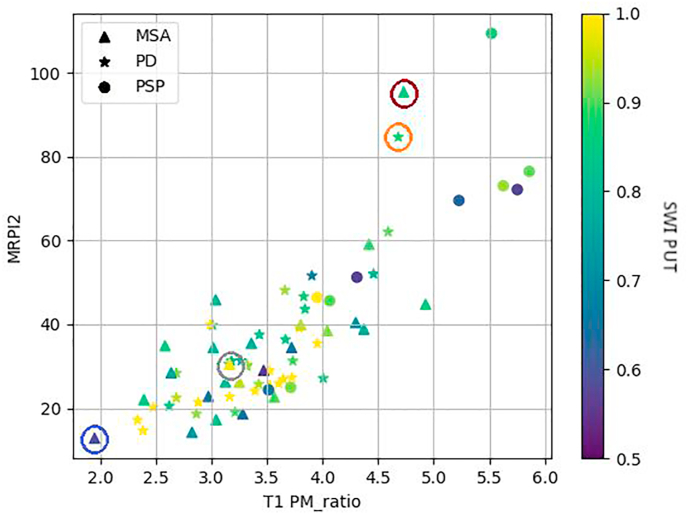
Scatter plot of the T1 PM_ratio versus MRPI2 and scaled-colored according to the SWI values in the putamen with triangles for multiple system atrophy (MSA), stars for Parkinson's disease (PD), and spheres for progressive supranuclear palsy (PSP).

Fig. 4 shows the scatter plot of the three selected MR biomarkers for the three different diagnoses. We consider as outliers those patients who visually are far from other patients with the same diagnosis in at least one of the biomarkers. The plot shows four possible outliers circled in purple (outlier 1), with higher MRPI2 than the rest of MSA patients; orange (outlier 2), with higher MRPI2 than other PD patients; gray (outlier 3), with higher SWI PUT than other MSA patients with similar MRPI2 and T1 PM_ratio; and blue (outlier 4), with lower MRPI2 and T1 PM_ratio than other MSA patients.

Three out of these four outliers (cases 1, 2, and 3) were misclassified using Bayesian Inference, as shown in Table 6. For the outlier 1 there is still some doubt about the clinical diagnosis between MSA and PSP, but MSA was clinically more likely and the model classifies this case as PSP. For the outliers 2 and 3 there is no doubt about the clinical diagnosis. For the outlier 4 the clinical diagnosis is MSA, which is also how the model classified this case, but there is some clinical doubt of possible PD diagnosis.

**Table 6:** Summary of the results of the Bayesian Inference classification for the outliers observed as outliers in the scatter plot (Fig. 4).

Case	Final clinical Diagnosis	MSA (%)	PD (%)	PSP (%)	Predicted	Age	Gender
Outlier 1	MSA	00.01	00.08	99.91	False	76	Male
Outlier 2	PD	01.36	00.44	98.20	False	79	Female
Outlier 3	MSA-C	35.25	52.15	12.60	False	58	Male
Outlier 4	MSA	58.94	01.80	39.27	True	55	Female

## 6 Discussion

In this study, we applied a previously proposed (Seada et al., 2023) decision model based on quantitative MRI biomarkers on an available data set to explore its potential. We automatize the process without human interaction, therefore, the classification was blind to patient's known follow up. The strength of this study was its innovative approach, namely by including people only with an uncertain diagnosis at the time of scanning. These baseline results were compared to a silver standard diagnosis that was obtained after follow-up, based on rate of progression, development of additional red flags, and the treatment response. This allowed us to ascertain the value of neuroimaging performed at a time when it was needed most, namely when the clinician had diagnostic uncertainty. The model correctly classified 59 % of patients (43 out of 73) into their final clinical diagnosis of PD, MSA, or PSP that was obtained at follow-up. These results suggest potential for quantitative MRI in improving diagnostics in the earliest stages of the disease, at a time when clinicians need ancillary testing to reduce their clinical uncertainty. Being able to establish an accurate diagnosis earlier in the disease course is important for many reasons, in particular to offer affected individuals their actual diagnosis and, within margins, an estimate of their possible prognosis. A timely diagnosis is also helpful to raise early awareness for specific disease complications, such as nocturnal sudden death due to stridor in patients with MSA. And an early accurate diagnosis helps with including the right patients for the right trials.

The 8-rules method was reduced to the three most useful rules for different reasons. First, the two initial rules are used to distinguish between healthy and non-healthy subjects. Healthy controls were not included in this study since this distinction is not so challenging in clinical practice. Secondly, we decided to discard the rules based on DTI (4 and 7) because ANOVA analyses showed no significant differences between PD, MSA, and PSP. Finally, we didn't include rule 8, which is focused on distinguishing between MSAp and MSAc, because the number of patients with MSAc was too low.

The lack of differences between patient groups in DTI biomarkers contrasts previous findings in the literature (Du et al., 2017; Worker et al., 2014). One reason for this could be that we only used fractional anisotropy, while previous studies indicate possible benefits from adding other DTI-related measurements, including diffusion anisotropy, radial diffusivity, longitudinal diffusivity, and mean diffusivity in different regions of interest) to improve classification (Planetta et al., 2016; Prodoehl et al., 2013) or DTI in combination with other measurements as apparent transverse relaxation (Du et al., 2017). However, the results are inconsistent (Min et al., 2018). Also, the segmentation of the peduncles is very challenging since they are tiny structures and a relatively thick slice-thickness (5 mm) can hinder a correct segmentation. In future studies, it could be useful to increase the resolution and to include more DTI-biomarkers and evaluate if the combination of them helps to improve the classifications.

We used two different models: model I performs one single multi-classification with all the biomarkers available, whereas model II uses a two-step classification where patients are first classified as PSP or no PSP and then the latter group is further classified as PD or MSA. Our results show a slightly better performance of model I. In this case, it could be because there are differences in atrophy between PD and MSA, therefore, using them as a single group to compare with PSP makes the differences less obvious than separately comparing PSP vs PD and PSP vs MSA (Quattrone et al., 2008; Möller et al., 2017; Mangesius et al., 2018). The estimations showed good specificity but lower sensitivity for classification and better accuracy for PSP than the rest. One explanation for this is the fact that patterns of atrophy are most distinct for PSP (Quattrone et al., 2008, 2018; Möller et al., 2017; Mangesius et al., 2020), while atrophy measurements for PD and MSA greatly overlap and thus it is harder to differentiate from each other. Moreover, due to the low number of patients with MSA-C we had to lump all MSA cases together for the analyses, which could have led to lower accuracy. For instance, atrophy was reported to distinguish PD from MSAc (Möller et al., 2017). Finally, rule 6 is supposed to use QSM-based iron concentrations to differentiate between MSA and PD (Mazzucchi et al., 2019; Sjöström et al., 2017) but here we used non-quantitative SWI which could be less robust and hamper a proper classification (Pyatigorskaya et al., 2020a). Iron concentration (either using SWI or QSM) has been reported as a helpful biomarker (Pyatigorskaya et al., 2020a; Meijer et al., 2015, 2016; Gupta et al., 2010), specially evident in MASp patients (Meijer et al., 2015; Hwang et al., 2015). Some overlap can be noted between patients groups since these susceptibility changes of the lentiform nucleus can be seen in several disorders, and even as part of normal ageing. However, the combination of these susceptibility changes with putaminal atrophy is diagnostically specific for MSAp. This highlight the importance of an integrated assessment of imaging findings (Chung et al., 2009) as we demonstrated here using SWI in combination with atrophy to classify PD, MSA, and PSP. We also hypothesize that the use of QSM instead of SWI would improve the accuracy and robustness of the method overall in differentiating between MSA and PD patients.

The accuracy can also be limited by the simplicity of the classification model (Bayesian Inference), which could be improved by the use of DeepLearning models where more characteristics and properties could be considered to infer the actual diagnosis. However, to perform such an analysis much more patients than the current cohort are needed (at least 30 patients per group). The recruitment and assessment of more patients towards a more precise model is encouraged by the insights obtained in this pilot study.

Regarding the four outliers that we depicted, 3 of them were incorrectly classified. Outliers 1 and 2 have diagnoses as MSA and PD respectively, but our classifier detects it as PSP. Both have higher MRPI2 than the MSA or PD group, which has been shown as characteristic in PSP patients (Quattrone et al., 2008, 2018). In the follow-up, however, there are only doubts about the clinical diagnosis for the first case. Outlier 3 has a higher SWI in putamen than expected, which is associated with PD and it is how our model is classifying it. After reviewing the case by our specialist, it has the same diagnosis but follow-up would be recommended. The progression of the disease in these cases would be interesting feedback to further evaluate the performance of this model. The last case (oulier 4) is correctly classified. We pointed it as an outlier for having smaller values than the rest, but it can be also that it is not an actual outlier since the proportions are coherent (lower T1 PM_ratio with lower MRPI2 and lower SWI in the putamen), therefore the biomarkers are statistically similar to the rest of MSA patients (Fig. 4) and it is correctly classified.

This study has two major strengths; one is the inclusion of biomarkers obtained from different types of images; and the other is that the MRI scans were acquired at an early stage of the disease at a time when the clinical diagnosis was uncertain. Most previous studies are based on patients with clear diagnoses and thus those conclusions may not be applicable to the early stages. Moreover, patients had clinical follow-ups, probably increasing the accuracy of the final clinical diagnosis. However, as stated before, even in the late stages of the disease there is a high rate of misdiagnoses (Hughes et al., 2002; Joutsa et al., 2014). It is possible that misclassified patients, such as outliers 1–3, actually have a different pathological diagnosis. Thus, a study with pathological confirmation of diagnoses through autopsy would be ideal, but it would be even more difficult to recruit sufficient numbers for adequate analyses.

## 7 Conclusion

In summary, we applied a quantitative MRI-based decision model to a cohort of patients with PD, MSA, and PSP who had undergone MRI in the early stage of the disease when clinical diagnosis was uncertain. We showed that a combination of atrophy and SWI biomarkers can correctly classify 59 % of patients into their final clinical diagnosis. These results are very promising and urge further studies with a higher number of patients per diagnosis and additional MR biomarkers to optimize and validate the decision model.

## Code Availability

The scripts used in this study were customized to our systems and files to automatize the tools mentioned in the ‘Methods’ section and facilitate our processing. However, it doesn't include any novelty. Hence the code is not disclosed publicly.

## CRediT authorship contribution statement

**Laura Nunez-Gonzalez:** Writing – review & editing, Writing – original draft, Visualization, Validation, Software, Methodology, Investigation, Formal analysis, Data curation, Conceptualization. **Elise G.P. Dopper:** Writing – review & editing, Visualization, Resources, Methodology, Investigation. **Anke W. van der Eerden:** Writing – review & editing, Visualization, Resources, Methodology, Investigation, Funding acquisition, Conceptualization. **Samy Abo Seada:** Writing – review & editing, Visualization, Validation, Software, Methodology, Investigation, Funding acquisition, Conceptualization. **Agnita J.W. Boon:** Writing – review & editing, Visualization, Methodology, Conceptualization. **Marcel M. Verbeek:** Writing – review & editing, Validation, Supervision, Resources, Investigation, Funding acquisition, Data curation. **Bastiaan R. Bloem:** Writing – review & editing, Validation, Supervision, Resources, Investigation, Funding acquisition, Data curation. **Frederick Jan Anton Meijer:** Writing – review & editing, Visualization, Validation, Resources, Methodology, Investigation, Data curation, Conceptualization. **Juan Antonio Hernandez-Tamames:** Writing – review & editing, Visualization, Validation, Supervision, Resources, Project administration, Methodology, Investigation, Funding acquisition, Conceptualization.

## Declaration of Competing interest

Prof. Bloem serves as the co-Editor in Chief for the Journal of Parkinson's disease, serves on the editorial board of Practical Neurology and Digital Biomarkers, has received fees from serving on the scientific advisory board for the Critical Path Institute, Gyenno Science, MedRhythms, UCB, Kyowa Kirin and Zambon (paid to the Institute), has received fees for speaking at conferences from AbbVie, Bial, Biogen, GE Healthcare, Oruen, Roche, UCB and Zambon (paid to the Institute), and has received research support from 10.13039/100005614Biogen, Cure Parkinson's, 10.13039/100009862Davis Phinney Foundation, Edmond J. Safra Foundation, Fred Foundation, Gatsby Foundation, Hersenstichting Nederland, Horizon 2020, IRLAB Therapeutics, Maag Lever Darm Stichting, Michael J Fox Foundation, Ministry of Agriculture, Ministry of Economic Affairs & Climate Policy, Ministry of Health, Welfare and Sport, Netherlands Organization for Scientific Research (ZonMw), Not Impossible, Parkinson Vereniging, Parkinson's Foundation, Parkinson's UK, Stichting Alkemade-Keuls, Stichting Parkinson NL, Stichting Woelse Waard, Health Holland/Topsector Life Sciences and Health, UCB, Verily Life Sciences, Roche and Zambon.

The other authors don't declare any conflict of interest.

## Data Availability

The data that has been used is confidential.
