# Supplementary material for: Quantitative MRI-based decision model for early-stage parkinsonism diagnosis: a pilot feasibility study
Source: Neuroimage Rep. 2025 Jun 14;5(3):100273. doi: 10.1016/j.ynirp.2025.100273 (PMC12489762; doi:10.1016/j.ynirp.2025.100273)
Supplement: Multimedia component 1 [file mmc1.doc]

SUPPLEMENTARY DATA

| **Patient** | **MSA (%)** | **PD (%)** | **PSP (%)** | **Predicted** | **Age** | **Gender** | **Disease**  **Duration**  **(months)** | **MMSE**  **score** | **HY**  **stadium** | **UPDRS**  **score** | **Follow-up**  **(months)** |
| --- | --- | --- | --- | --- | --- | --- | --- | --- | --- | --- | --- |
| 005 | 35.67 | 47.69 | 16.64 | True | 61 | Male | 36 | 30 | 2 | 46 | 42 |
| 010 | 13.43 | 27.74 | 58.83 | False | 73 | Male | - | - | - | - | 33 |
| 011 | 32.26 | 60.96 | 06.78 | True | 72 | Female | - | - | 1 | - | 40 |
| 014 | 46.65 | 37.02 | 16.34 | False | 61 | Male | 24 | 29 | 1 | 13 | 24 |
| 015 | 23.43 | 54.38 | 22.19 | True | 65 | Female | 10 | 29 | 3 | 43 | 4 |
| 017 | 64.67 | 28.70 | 06.63 | False | 64 | Male | 9 | 29 | 1 | 18 | 25 |
| 020 | 39.68 | 42.24 | 18.08 | True | 58 | Male | 6 | 29 | 2 | 23 | 30 |
| 022 | 28.79 | 44.63 | 26.58 | True | 55 | Male | - | - | 2 | - | 42 |
| 024 | 01.36 | 00.44 | 98.20 | False | 79 | Female | 18 | 26 | 2 | 54 | 27 |
| 025 | 28.11 | 63.49 | 08.39 | True | 67 | Female | 24 | 27 | 3 | 37 | 31 |
| 026 | 40.84 | 52.97 | 06.19 | True | 76 | Female | - | 29 | - | - | 36 |
| 028 | 33.11 | 60.93 | 05.96 | True | 62 | Male | - | 30 | 2 | 20 | 41 |
| 032 | 35.23 | 57.24 | 07.52 | True | 76 | Female | 12 | 29 | 3 | 49 | 30 |
| 034 | 56.25 | 16.30 | 27.45 | False | 57 | Male | 15 | 29 | 1 | 31 | 24 |
| 035 | 47.32 | 47.89 | 04.79 | True | 50 | Male | 24 | 27 | 2 | 44 | 5 |
| 036 | 36.91 | 57.68 | 05.41 | True | 55 | Female | 36 | 30 | 2 | 32 | 13 |
| 040 | 53.00 | 22.87 | 24.13 | False | 42 | Female | - | - | - | - | 36 |
| 043 | 54.43 | 36.84 | 08.73 | False | 53 | Male | 16 | 27 | 1 | 19 | 36 |
| 044 | 23.97 | 63.45 | 12.58 | True | 75 | Male | - | - | - | - | 37 |
| 046 | 46.90 | 48.30 | 04.81 | True | 73 | Male | 24 | 29 | 1 | 41 | 12 |
| 050 | 41.15 | 53.19 | 05.66 | True | 56 | Male | 48 | 25 | 2 | 42 | 2 |
| 052 | 29.67 | 52.31 | 18.02 | True | 50 | Male | - | - | - | - | 38 |
| 053 | 36.00 | 51.71 | 12.29 | True | 52 | Female | 30 | 29 | 2 | 31 | 24 |
| 055 | 27.81 | 61.01 | 11.18 | True | 59 | Male | - | - | 2 | - | - |
| 057 | 32.99 | 50.28 | 16.72 | True | 57 | Male | 12 | 29 | 1 | 33 | 31 |
| 061 | 37.12 | 54.76 | 08.12 | True | 44 | Female | - | - | - | - | 36 |
| 063 | 34.71 | 56.25 | 09.04 | True | 72 | Female | 24 | 30 | 2 | 34 | 27 |
| 066 | 49.13 | 44.21 | 06.66 | False | 52 | Male | 44 | 28 | 2 | 30 | 2 |
| 074 | 36.87 | 56.46 | 06.67 | True | 56 | Male | 36 | 30 | 2 | 40 | 32 |
| 075 | 44.17 | 34.30 | 21.53 | False | 72 | Male | - | - | - | - | - |
| 083 | 25.33 | 49.04 | 25.63 | True | 58 | Female | 36 | 30 | 1 | 22 | 36 |
| 084 | 56.22 | 15.13 | 28.65 | False | 53 | Male | 27 | 24 | 1 | 26 | 35 |
| 087 | 31.75 | 50.81 | 17.44 | True | 60 | Female | 19 | 30 | 2 | 33 | 28 |
| 089 | 21.67 | 47.86 | 30.47 | True | 61 | Female | 42 | - | - | - | 22 |
| 094 | 51.03 | 40.93 | 08.04 | False | 59 | Male | - | 26 | - | - | 36 |
| 097 | 53.44 | 35.81 | 10.75 | False | 75 | Female | - | 30 | 2 | 36 | 52 |
| 103 | 31.68 | 54.99 | 13.33 | True | 61 | Female | 18 | 28 | 1 | 16 | 4 |
| 105 | 24.52 | 62.73 | 12.75 | True | 62 | Male | 24 | 28 | 1 | 30 | 2 |

*Table S1. Results of the Bayesian Inference using the model I for patients with Parkinson’s Disease (PD). Disease duration is the time from the first symptoms to the time of inclusion to perform the MRI scan. The rows where the model predicts the diagnosis correctly are written In green letters and in black the ones where the model prediction doesn’t match the actual diagnosis. The highest percentages for the latest are shown in red letters.*

| **Patient** | **MSA (%)** | **PD (%)** | **PSP (%)** | **Predicted** | **Age** | **Gender** | **Disease**  **Duration (months)** | **MMSE score** | **HY stadium** | **UPDRS score** | **Follow-up**  **(months)** |
| --- | --- | --- | --- | --- | --- | --- | --- | --- | --- | --- | --- |
| 002* | 14.59 | 45.97 | 39.44 | False | 68 | Male | 13 | 30 | 2 | 29 | 26 |
| 008* | 23.04 | 65.01 | 11.95 | False | 71 | Male | - | - | - | - | - |
| 013*^+^* | 74.32 | 16.79 | 08.88 | True | 52 | Female | 36 | 30 | 2 | 51 | 5 |
| 016 | 41.91 | 52.88 | 05.21 | False | 78 | Female | - | 26 | - | - | - |
| 018* | 38.23 | 53.33 | 08.44 | False | 57 | Female |  | - - | 2 | - | - |
| 019* | 58.49 | 30.06 | 11.45 | True | 63 | Male | 21 | 28 | 3 | 49 | 3 |
| 021* | 64.55 | 29.26 | 06.19 | True | 73 | Male | - | - | 3 | - | - |
| 023 | 38.92 | 54.46 | 06.62 | False | 53 | Male | - | - | 2 | - | 36 |
| 029 | 33.20 | 52.90 | 13.90 | False | 59 | Female | 06 | 30 | 2 | 30 | 19 |
| 031 | 31.78 | 54.59 | 13.63 | False | 69 | Female | - | 25 | 2 | 50 | 36 |
| 033* | 40.21 | 43.35 | 16.44 | False | 68 | Male | 48 | 30 | 3 | 55 | 8 |
| 042 | 58.94 | 01.80 | 39.27 | True | 55 | Female | - | - | - | - | - |
| 045* | 17.23 | 40.04 | 42.73 | False | 63 | Male | 36 | 28 | 2 | 39 | 26 |
| 051 | 00.01 | 00.08 | 99.91 | False | 76 | Male | - | - | - | - | - |
| 056 | 74.06 | 18.80 | 07.14 | True | 62 | Male | - | - | 3 | - | 41 |
| 058 | 74.08 | 15.55 | 10.37 | True | 57 | Male | - | - | - | - | - |
| 059* | 42.37 | 50.23 | 07.40 | False | 75 | Female | - | - | - | - | - |
| 060* | 65.57 | 07.28 | 27.14 | True | 63 | Female | - | - | - | - | - |
| 068* | 46.46 | 48.36 | 05.18 | False | 80 | Female | 21 | 29 | 3 | 47 | 2 |
| 069*^+^* | 35.25 | 52.15 | 12.60 | False | 58 | Male | 26 | 28 | 3 | 45 | 20 |
| 076* | 23.13 | 65.54 | 11.32 | False | 63 | Male | - | - | - | - | - |
| 078* | 49.40 | 45.78 | 04.82 | True | 63 | Male | - | - | - | - | 39 |
| 088* | 52.41 | 40.39 | 07.19 | True | 69 | MAle | 12 | 27 | 2 | 51 | 27 |
| 090 | 58.52 | 35.86 | 04.61 | True | 54 | Male | - | - | - | - | - |
| 101 | 65.84 | 26.26 | 07.90 | True | 56 | Male | - | 28 | 2 | 22 | 35 |

*Table S2. Results of the Bayesian Inference using model I for patients with unclassified multiple system atrophy (MSA). ^+^cerebellar multiple system atrophy (MSA-C).*Parkinsonian multiple system atrophy (MSA-P).* *Disease duration is the time from the first symptoms to the time of inclusion to perform the MRI scan.Results of the Bayesian Inference using the model I for patients with Parkinson’s Disease (PD). Disease duration is the time from the first symptoms to the time of inclusion to perform the MRI scan. The rows where the model predicts the diagnosis correctly are written In green letters and in black the ones where the model prediction doesn’t match the actual diagnosis. The highest percentages for the latest are shown in red letters.*

| **Patient** | **MSA (%)** | **PD (%)** | **PSP (%)** | **Predicted** | **Age** | **Gender** | **Disease**  **Duration**  **(months)** | **MMSE**  **score** | **HY**  **stadium** | **UPDRS**  **score** | **Follow-up**  **(months)** |
| --- | --- | --- | --- | --- | --- | --- | --- | --- | --- | --- | --- |
| 001 | 00.42 | 00.34 | 99.24 | True | 61 | Female | 48 | 29 | 3 | 38 | 33 |
| 003 | 35.29 | 57.87 | 06.84 | False | 55 | Male | - | 28 | 2 | 21 | 49 |
| 027 | 00.15 | 00.07 | 99.79 | True | 68 | Female | 30 | 28 | - | - | 10 |
| 039* | 23.26 | 65.41 | 11.32 | False | 58 | Male | - | - | - | - | - |
| 067 | 18.82 | 52.20 | 28.99 | False | 68 | Male | - | - | - | - | 48 |
| 080 | 00.00 | 00.00 | 100.00 | True | 71 | Male | 36 | 29 | 3 | 57 | 36 |
| 092 | 02.41 | 00.44 | 97.15 | True | 66 | Male | - | 28 | 2 | 21 | - |
| 093 | 00.30 | 00.01 | 99.69 | True | 63 | Male | - | - | 3 | - | - |
| 096 | 35.56 | 06.76 | 57.68 | True | 70 | Male | - | 27 | 3 | 16 | - |
| 099*^+^* | 68.69 | 24.95 | 06.36 | False | 63 | Female | - | 30 | 3 | 31 | - |

*Table S3. Results of the Bayesian Inference using the model I for patients with progressive supranuclear palsy (PSP). * PSP (Richardson). ^+^PSP-P. Disease duration is the time from the first symptoms to the time of inclusion to perform the MRI scan.Results of the Bayesian Inference using the model I for patients with Parkinson’s Disease (PD). Disease duration is the time from the first symptoms to the time of inclusion to perform the MRI scan. The rows where the model predicts the diagnosis correctly are written In green letters and in black the ones where the model prediction doesn’t match the actual diagnosis. The highest percentages for the latest are shown in red letters.*

*
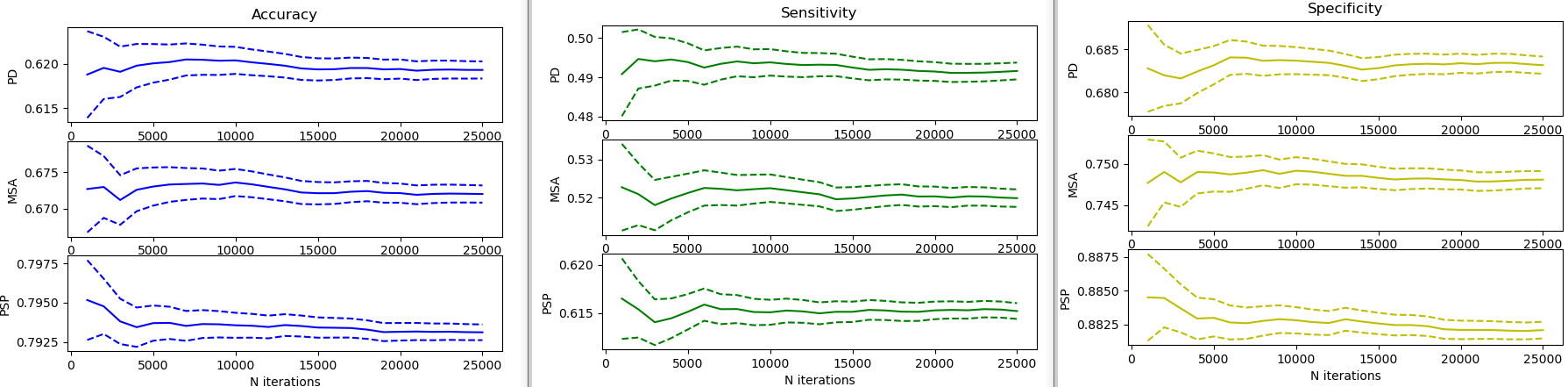
*

*Figure S1. Accuracy (left), sensitivity (middle), and specificity (right) when classifying Parkinson’s disease (PD), multiple system atrophy (MSA), and progressive supranuclear palsy (PSP) using the model I for different numbers of random-sampling iterations during the bootstrapping (N iterations).*
